# COVID-AleXception: A Deep Learning Model Based on a Deep Feature Concatenation Approach for the Detection of COVID-19 from Chest X-ray Images

**DOI:** 10.3390/healthcare10102072

**Published:** 2022-10-18

**Authors:** Manel Ayadi, Amel Ksibi, Amal Al-Rasheed, Ben Othman Soufiene

**Affiliations:** 1 Department of Information Systems, College of Computer and Information Sciences, Princess Nourah bint Abdulrahman University, P.O. Box 84428, Riyadh 11671, Saudi Arabia; 2 Prince Laboratory Research, ISITcom (Institut Supérieur d’Informatique et des Techniques de Communication de Hammam Sousse), University of Sousse, Hammam Sousse 4023, Tunisia

**Keywords:** deep learning, COVID-19, chest X-ray images, AlexNet, Xception, deep feature concatenation, COVID-AlexCeption

## Abstract

The novel coronavirus 2019 (COVID-19) spread rapidly around the world and its outbreak has become a pandemic. Due to an increase in afflicted cases, the quantity of COVID-19 tests kits available in hospitals has decreased. Therefore, an autonomous detection system is an essential tool for reducing infection risks and spreading of the virus. In the literature, various models based on machine learning (ML) and deep learning (DL) are introduced to detect many pneumonias using chest X-ray images. The cornerstone in this paper is the use of pretrained deep learning CNN architectures to construct an automated system for COVID-19 detection and diagnosis. In this work, we used the deep feature concatenation (DFC) mechanism to combine features extracted from input images using the two modern pre-trained CNN models, AlexNet and Xception. Hence, we propose COVID-AleXception: a neural network that is a concatenation of the AlexNet and Xception models for the overall improvement of the prediction capability of this pandemic. To evaluate the proposed model and build a dataset of large-scale X-ray images, there was a careful selection of multiple X-ray images from several sources. The COVID-AleXception model can achieve a classification accuracy of 98.68%, which shows the superiority of the proposed model over AlexNet and Xception that achieved a classification accuracy of 94.86% and 95.63%, respectively. The performance results of this proposed model demonstrate its pertinence to help radiologists diagnose COVID-19 more quickly.

## 1. Introduction

On 31 December 2019, the World Health Organization (WHO) received notification of a novel coronavirus [1]. The disease’s bothersome characteristics are its ease of transmission and asymptomatic presentation, which can be a source of development [2]. Dry cough, sore throat, and fever are the most prevalent COVID-19 symptoms. Septic shock, pulmonary edema, acute respiratory distress syndrome, and multi-organ failure can all occur if symptoms progress to a severe type of pneumonia. With reference to an update announced by the WHO on the 9 October 2022, 626,500,862 COVID-19 cases were confirmed in which there were 6,560,744 deaths [3]. Because no medical treatment is available, the prompt diagnosis of COVID-19 is required to prevent further spread and to treat the patient in a timely manner. In the beginning, the technique for the detection of this pandemic was RT-PCR, which was the first available technique to combat this worldwide spread. Because of the high demand for test kits in many parts of the world, fresh corona pneumonia screening is delayed, resulting in the virus’s spread [4]. So, it is necessary to develop an intelligent predictive model of COVID-19 based on machine learning methods for corrective measures following the clinician decisions. Apart from clinical procedures, researchers have shown that artificial intelligence and machine learning are promising technologies adopted by a variety of health care providers due to their high potential to scale-up and speed-up processing power, their reliability, as well as outperforming humans in certain healthcare tasks [5].

Even though if negative results are observed, they should not be treated as definitive due to the poor sensitivity value of RT-PCR, which has 60% to 70% accuracy. On top of that, symptoms can be diagnosed and detected with the aid of using the patient’s radiographic images [6]. Chest radiological imaging, mainly comprising X-ray and computed tomography (CT), play a significant role not only in the prompt diagnosis but also in the treatment of COVID-19 [7]. As a result, it is important to merge these radiographic pictures with an artificial intelligence system for more accurate COVID-19 forecasts. Deep CNN is the deep learning mechanism that offers enormous success when compared to other conventional methods in different image complexities [8]. Deep learning has enabled researchers to solve various complex problems. It has also shown a high performance not only in vision but also in machine learning tasks such as image classification, speech and voice recognition, natural language processing, object detection, medical imaging, etc. [9].

Due to the importance of this topic, numerous approaches have recently been proposed to classify COVID-19 using chest X-ray images. Anichur et al. [10] utilized a deep learning model to diagnose COVID-19 via X-ray images and proposed a COVIDX-Net model comprising seven CNN models. Sujata et al. [11] proposed a deep learning model for the detection of COVID-19 (COVID-Net) with an accuracy of 92.4% in classifying three classes that include normal (non-COVID), pneumonia and COVID-19. In another study, Muhammad et al. [12] developed a deep learning model which classified COVID-19 infection, as well as normal and pneumonia classes. They used 224 images with confirmed COVID-19 cases. Their model achieved a success rate of 98.75% and 93.48% for two and three classes, respectively. Kumar et al. [13] developed a custom-made deep learning architecture named SARS-Net to classify and detect the Chest X-ray images for COVID-19 diagnosis. Their model achieved an accuracy of 97.60% and a sensitivity of 92.90% on the validation set. To classify the features obtained from different X-ray images, Zouch et al. [14] used the convolutional neural network (CNN) models together with the support of vector machine (SVM) classifier. 

In this paper, a hybrid deep learning model for the prediction of COVID-19 from chest X-ray images is proposed. This model is a concatenation between AlexNet and Xception models named COVID–AlexCeption. The combination of publicly open datasets in the experimental studies has enabled us to obtain a set of 15,153 X-ray images that contain normal (healthy), COVID-19, and Pneumonia classes. The COVID-AleXception model can achieve a classification accuracy of 98.68% that shows the superiority of the proposed model over AlexNet and Xception, which achieved a classification accuracy of 94.86% and 95.63% respectively.

Our main contributions can be summarized as follows:

❖ The collection of a medical X-ray image dataset includes three main classes (normal, pneumonia, and COVID-19) for the training and testing of the proposed system.
❖ To perform a DFC technique in order to benefit from combining deep features which were extracted from AlexNet and Xception models.
❖ We propose COVID-AlexCeption: a deep learning model with concatenation of AlexNet and Xception models to detect COVID-19 from X-ray images.
❖ The performance of COVID-AlexCeption has been tested.
❖ The comparison of COVID-AlexCeption to competitive methods in terms of different performance metrics has been performed, mainly in terms of f-measure, accuracy, precision, and recall.

The remainder of our paper is organized this way: related works are investigated in Section 2. Materials and Methods are presented in detail in Section 3. In Section 4, we describe the implementation and testbed, followed by the experimental results in Section 5. Finally, conclusions are presented in Section 5.

## 2. State-of-the-Art Methods

With the purpose of identifying persons infected with COVID-19, a variety of works use transfer learning on chest X-ray images. The different works presented in this section are summarized in Table 1. 

The work presented in [15] shows an algorithm, nCOVnet, that is used to detect COVID-19 patients making use of X-ray images. This system is meant to help determine whether a person is actively infected by coronavirus or not. The forecast model consists of 24 layers. The first layer represents the input layer. An input RGB image with 224 × 224 × 3 pixels dimensions has been fixed. The next 18 layers represent a combination of the convolution layers plus ReLU (Rectified Linear Unit) and Max Pooling Layers. Then, a transfer learning model was applied using the five different layers that were proposed and trained. The fundamental structure of the network of this proposed model is based upon four layers, which are the convolution layer, aggregation layer, flattening and fully connected layers. The authors made use of 337 images in order to evaluate the system. The suggested nCOVnet model efficiently detects patients infected with COVID-19 with 97% accuracy, which represents an encouraging result compared to previous models. 

Belal Hossain et al. [16] developed a transfer learning with fine-tuned deep CNN ResNet50 model for classifying COVID-19 from chest X-ray images. In this paper, the authors present a method for how to identify the presence of COVID-19 in X-ray images, using transfer learning (TL) on a ResNet50 model. In this work, the authors used 10 different pre-trained weights. The 10 pre-trained models are: ChestX-ray14, ChexPert, ImageNet, *ImageNet_C* h *estX—ray*14, *ImageNet_C* h *exPert*, *iNat*2021*_Supervised*, *iNat*2021*_Supervised_Form_Scratch*, *iNat*2021*_Mini_SwAV _*1*k*, and *MoCo_v*1, *MoCo_v*2. The main goal of this system was detecting whether a person is a carrier of the virus or normal. The proposed model correctly detects COVID-19 with 99.17% validation accuracy, 99.95% training accuracy, 99.31% precision, 99.03% sensitivity, and 99.17% F1-score.

To detect COVID-19, Sakshi et al. [17] deployed a model making use of transfer learning from CT scan images divided into three levels with the use of stationary wavelets. Three major steps make the basis of this proposed model. Step one is data augmentation. Due to the shortage of databases, the number of the processed images is augmented to three levels, making use of fixed waves where shear, rotation and transition have been applied to all those images. Step two is detecting COVID-19 using pre-trained CNN models. In this step, CT images are classified into two categories (COVID+ and COVID−), making use of the techniques of learning-based transmission. To do this task, four pre-trained transfer learning models, ResNet18, ResNet101, ResNet50 and SqueezeNet, have been used. Later on, the performance of the different models was compared to find out the best. Step three is anomaly localization. In this step, the feature map together with the activation layers of the best transmission learning models that were extracted from step two are deployed in order to translate the anomaly to the chest CT scan images of the positive cases of COVID-19. Results have shown that the highest classification accuracy was achieved through ResNet18 (training = 99.82%, validation = 97.32%, and testing = 99.4%). 

Likewise, the authors in reference [18] made use of four CNN architectures, which are VGG19-CNN, ResNet152V2, ResNet152V2 + Gated Recurrent Unit (GRU), and ResNet152V2 + Bidirectional GRU (Bi-GRU), in order to detect COVID-19 through the use of public digital chest X-ray plus CT datasets, having four classes (i.e., normal, COVID-19, pneumonia, and lung cancer). The first step handles the pre-processing of images such as resizing, image augmentation and the splitting of datasets that are randomly divided into 70% being used as a training set and 30% as a testing set. The second and third steps are feature extraction and image classification, respectively. Based on the findings of the experiments, it is clear that the VGG19 + CNN model has a better performance compared to the three other proposed models. The VGG19 + CNN model has led to an accuracy (ACC) of 98.05%, recall of 98.05%, precision of 98.43%, specificity (SPC) of 99.5%, negative predictive value (NPV) of 99.3% and F1 score of 98.24%. 

In [19], a diagnostic test was proposed by authors in order to detect COVID-19 cases. The rationale was to distinguish COVID-19 cases from bacterial pneumonia, viral pneumonia, and normal healthy people through the use of chest X-ray images and applying the deep learning neural networks ResNet50 and ResNet101. Cohen and Kaggle collected the two open-source image datasets. The proposed method was conducted in four major stages. The first stage consists of data visualization. The second stage is data augmentation. The third stage is data pre-processing while the fourth stage is deep neural network model designing, which is subdivided into stage-I and stage-II. In the image pre-processing phase, there are two sub-stages, which involve first finding out the minimum height and width of the dataset images and then resizing them according to the ImageNet database. In the second phase, which is data augmentation, the authors resorted to Rotation and Gaussian Blur in order to increase the number of images. Later on, the ResNet50 network is formed. In order to see the difference between viral pneumonia, bacterial pneumonia, and normal cases, this model has been trained. In stage-II, deep network model designing, the ResNet-101 network is formed. The first stage model presents a satisfactory performance which an accuracy reaching 93.01% in the identification of viral pneumonia, bacterial pneumonia, and healthy/normal people. The objective of the second stage model is to detect the existence of COVID-19. The findings in this stage show a significant performance with 97.22% accuracy. 

A new model to detect COVID-19 automatically using raw chest X-ray images was developed by Chiranjibi et al. [20]. It is considered as one of the first solutions that is based on the DL model (VGG-16) and the attention module. This solution is meant to provide dependable diagnostics not only for binary classification (COVID-19 and normal) but also multi-class classification (COVID-19, normal and pneumonia). This proposed method (also called attention-based VGG-16) consists of four main building blocks, such as an Attention module, Convolution module, FC-layers, and Softmax classifier. In this work, three COVID-19 CXR image datasets that are publicly available have been used. The created system achieved 79.58% accuracy. However, the performance of this proposed method could be further improved.

In reference [21], authors presented a deep convolutional neural-network-based architecture model to detect COVID-19, making use of chest radiography. The model is named FocusCOVID. The proposed model in this paper is end-to-end CNN architecture without any techniques of handcrafted feature extraction. In order to boost the performance of the model, residual learning and a squeeze-extinction network are employed by the proposed model. The two chosen datasets are Kaggle-1 and Kaggle-2. It includes 1143 COVID-19 specimens and 1345 normal and pneumonia radiograph specimens having a resolution of 1024 × 1024 and 256 × 256 pixels, respectively. The architecture involves a sum of 2,940,122 parameters. In the two-class classification (COVID-19, pneumonia and normal) for COVID-19 instances, the suggested FocusCOVID realized a 95.20% validation accuracy, a 96.40% train accuracy, a precision of 96.40%, a sensitivity of 95.20% and finally a 95.20% F1-score. 

CoroNet is proposed in [22]. It is a deep convolutional neural network model to detect COVID-19 infection automatically from chest X-ray images. The proposed CoroNet is based upon the Xception architecture which has been pre-trained in the ImageNet data set then trained end-to-end, making use of COVID-19 and chest pneumonia X-ray images extracted from two various publicly available databases. COVID-19, bormal, pneumonia–bacterial, and pneumonia–viral are the four categories on which CoroNet has been trained with the objective of classifying chest X-ray pictures. Researchers collected a number of 1300 images from these two available sources. Then, they dimensioned them to 224 × 224 pixels in size with a resolution of 72 dpi. CoroNet has 33,969,964 parameters in which 33,969,964 are trainable and 54,528 are non-trainable ones. On top of Tensorflow 2.0, CoroNet is implemented in Keras. As for four-class cases (COVID vs. pneumonia–bacterial vs. pneumonia–viral vs. normal), the proposed CoroNet achieved an 89.6% validation accuracy, a train accuracy of 90.40%, a precision of 90%, 96.40% sensitivity and an 89.9% F1-score.

In this study, in reference [23], researchers suggested a new architecture having the name CheXImageNet. This exclusive model presents an encouraging performance concerning the classification of COVID-19 with chest X-ray digital images. It is built upon using deep convolutional neural networks where the two used open-source image databases were obtained from Cohen and Kaggle. Pre-processing methods are applied in order to enhance the quality of images. During this process, merging datasets and resizing the images to 256 × 256 pixels is included. Once the data pre-processing method is applied, the data is split into training (80%) and testing (20%). The images are then classified into two distinct sets for binary class classification (normal cases and COVID-19 cases images) and into thee sets (COVID-19 cases, normal cases and images of pneumonia cases) for multi-class classification. The CheXImageNet architecture includes some layers and filters. The constituent of this architecture is: three convolutional layers, four batch normalization layers, three max pooling layers, seven LeakyReLU layers, five dense layers, one flattened layer, and four dropout layers. Authors claimed that 100% accuracy has been achieved for both binary classification (including COVID-19 cases and normal X-ray) and three-class classification (containing cases of COVID-19, normal X-ray, and cases of pneumonia) respectively. 

A new hybrid deep learning model is suggested in [24] by Ritika Nandi et al. It detects coronavirus from chest X-ray images. It is an amalgam of ResNet50 and MobileNet. These two models are used in a separate way then the results of each model are concatenated in order to produce the final results. This is followed by two fully joined layers. To update the neural network weights, the Adam optimization algorithm has been used. The suggested hybrid model’s performance was assessed on two publicly available COVID-19 chest X-ray data sets. Each of the datasets involves normal, pneumonia, and COVID-19 chest X-ray images. The results show that using the first dataset leads to an accuracy of 84.35%. However, using the second dataset leads to 94.43% accuracy. 

**Table 1:** Overview of studies using deep learning approaches for COVID-19 detection.

Ref	CNN Model	Data Sources	Accuracy (%)	Limitations
[15]	nCOVnet	Cohen et al. [25]	97.97	High execution time
[16]	ResNet50	Radiography Database [26]	99.17	Imbalanced data
[17]	ResNet18	CT scan images [27]	99.60	Overfitting issue
	ResNet50		99.20	
	ResNet101		99.30	
	SqueezeNet		99.50	
[18]	VGG19 +CNN	GitHub+ cancer X-ray and CT images [28]	98.05	Imbalanced data
	ResNet152V2		95.31	
	ResNet152V2 + GRU		96.09	
	ResNet152V2+ Bi-GRU		93.36	
[19]	ResNet50	Cohen [25] Kaggle [29]	93.01	Imbalanced data Overfitting issue
	ResNet101		97.22	
[20]	VGG-16	Khan et al. [22] Ozturk [20]	79.58	Overfitting issue
[21]	FocusCOVID	Kaggle-1 [30] Kaggle-2 [31]	95.20	Cannot provide the optimal accuracy.
[22]	CoroNet	Chest X-ray Images [32]	89.6	This method is slow.
[23]	CheXImageNet	Cohen [25] Kaggle [29]	100	Overfitting issue
[24]	ResNet50	Kaggle chest X-ray [33] RSNA pneumonia [34]	91.13	Cannot provide the optimal accuracy.
	MobileNet		93.73	
	Hybrid model		94.43	

## 3. Deep Learning Architectures

Deep learning (DL) is a type of machine learning algorithm that has lately shown tremendous promise in terms of computer vision and image processing [35]. Several deep learning models have been broadly utilized on chest X-ray pictures, generating excellent results in detection, segmentation, and classification tasks [26]. The AlexNet and Xception models are discussed in depth in this section.

### 3.1. AlexNet Model Architecture

**Figure 1:**
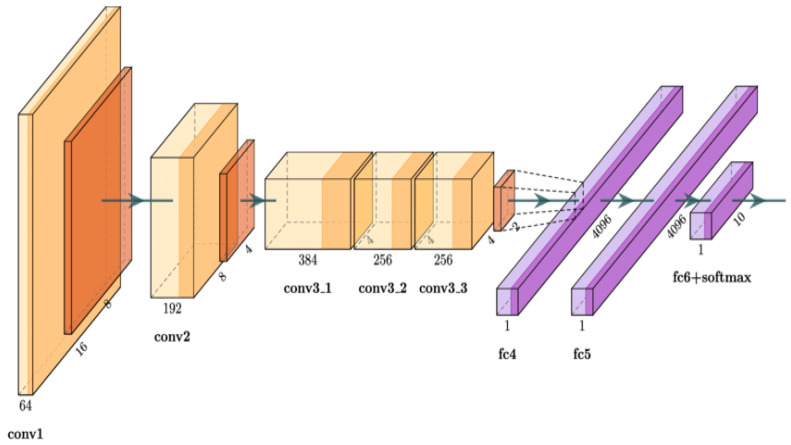
The architectures of the AlexNet model.

AlexNet architecture was developed by Alex Krishevesky et al. [35]. The architecture consists of eight layers: five convolutional layers and three fully connected layers. Figure 1 illustrates the basic design of the AlexNet architecture. The input picture of (224 × 224 × 3) is filtered using 96 kernels of size (11 × 11 × 3) in the first convolutional layer. The output of the first convolutional layer is sent into the second convolutional layer, which filters it using 256 kernels of size (5 × 5 × 48). The third convolution layer contains 384 kernels of size (3 × 3 × 256) connected to the outputs of the second convolutional layer (normalized, pooled). The fourth convolution layer contains 384 kernels of size (3 × 3 × 192), whereas the fifth convolution layer has 256 kernels of size (3 × 3 × 192). There are 4096 neurons in the fully linked layers. At the end and with dropout, two fully connected (FC) layers are employed, followed by a Softmax layer. This technique has been frequently utilized to analyze highly dimensional biomedical data automatically.

### 3.2. Xception Model Architecture

**Figure 2:**
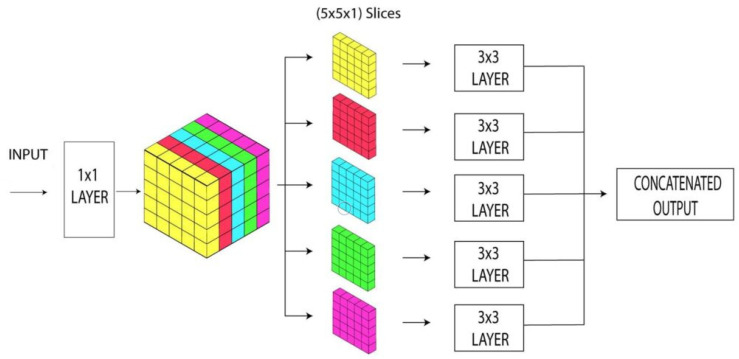
The architectures of the Xception model.

Francois [36] introduced the Xception architecture. It is an enhancement of the inception that takes the place of the regular inception modules, having distinguishable depth convolutions. The ultimate objective of the Xception architecture is to generate a network having more parameters which can be useful in solving the problems of any computer network. Figure 2 depicts the architecture of the Xception network model. Xception is based on two main phases. The depth-wise separable convolution is performed first, followed by a pointwise convolution. The channel-wise n × n spatial convolution is the depth-wise convolution. If the network comprises five channels, for example, we will get 5 n × n spatial convolution. 1 × 1 convolution is the pointwise convolution. As in residual networks, there are shortcuts between convolution blocks. Since Xception is a new model, there are only a few accessible cases to demonstrate its success [37].

## 4. Materials and Methods

**Figure 3:**
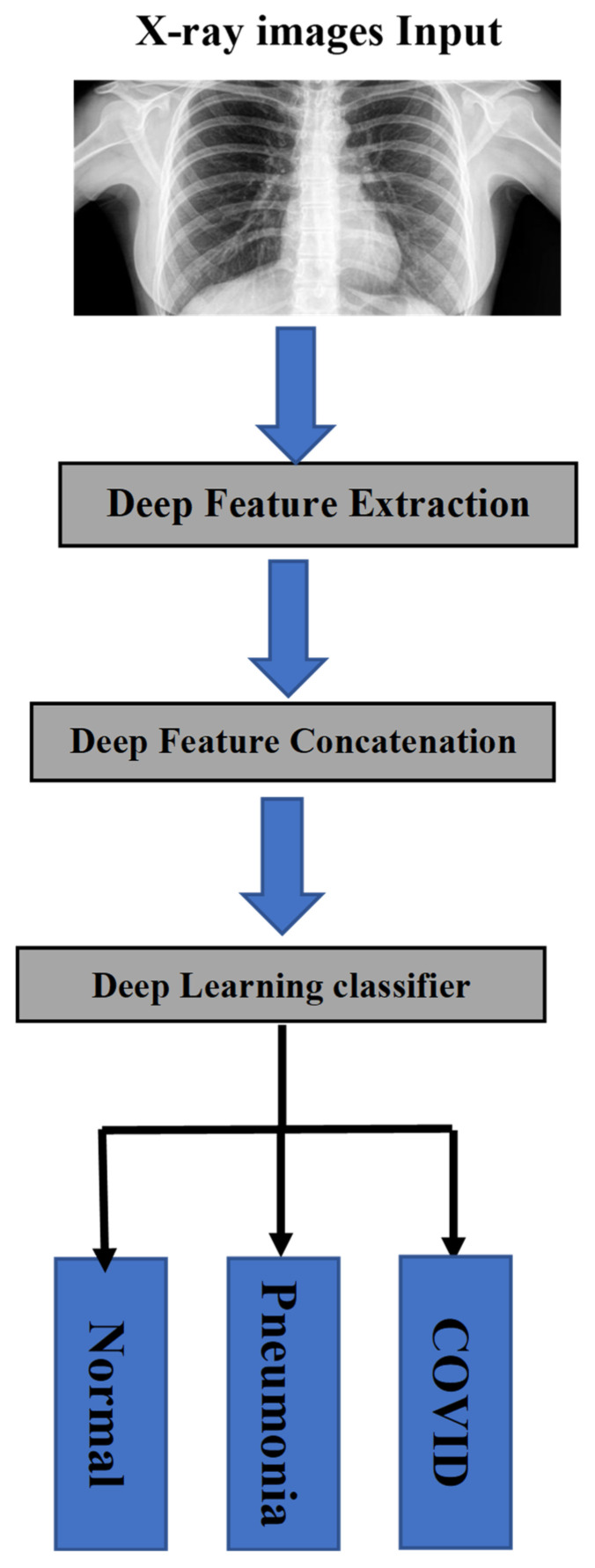
Simple block diagram of the proposed system.

In this section, the dataset as well as the block diagram of the proposed system are presented. Figure 3 gives a global overview of the methodology of the study, describing the deep learning proposed method built on deep feature concatenation. Firstly, (1) images were gathered from 5 COVID-19 radiography databases. (2) The pre-processing of images was done through resizing and normalization, a task that is going to be explained further. (3) Later on, data augmentation has been added with the purpose of solving the problem of overfitting resulting from the limited number of training images. Then, AlexNet and Xception deep learning models were used to extract the features from X-ray images. The DFC methodology was then applied in order to combine extracted features into one single descriptor to be classified. In this work, multiclass classification (COVID-19, normal, and pneumonia) was conducted with the proposed method. Below, there is an explanation of a detailed description of the architecture.

### 4.1. Dataset Description

**Figure 4:**
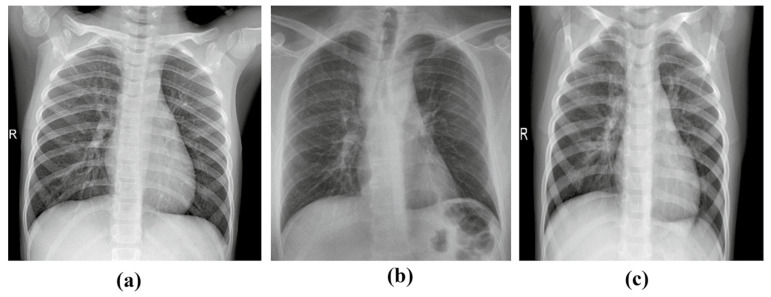
Samples of frontal-view chest X-ray images from the dataset: (**a**) normal case, (**b**) COVID-19 case, and (**c**) pneumonia.

Since COVID-19 is a newly discovered virus, the available number of COVID-19 X-ray images is limited and insufficient. In this proposed work, multiple X-ray images were carefully gathered from publicly available datasets to generate a large X-ray images dataset. 5 different open-source repositories were combined to form the dataset: (1) Cohen et al. [25], (2) Radiography Database [26], (3) Kaggle-1 [30], (4) Kaggle-2 [31] and (5) Chest X-ray Images [32]. These images were examined by experienced radiologists, who excluded any that lacked convincing COVID-19 evidence. Concerning the experimental analysis, 3 classes of chest X-ray images (normal, COVID-19, and pneumonia) have been considered. The dataset is composed of 15,153 X-ray images which are divided into 3 diseases as follows: 10,192 images of normal patients, 3616 X-ray images of COVID-19 and 1345 images of pneumonia. Figure 4 shows a sample of chest X-ray radiography image from every class. 

### 4.2. Data Preparation and Preprocessing

The input images are pre-processed using several pre-processing methodologies to improve the training process. The ultimate objective of pre-processing images is to enhance the visual capacity of the input image through the improvement of contrast, minimizing noise or eliminating low or high frequencies in the initial image. In this work, image resizing and image normalization pre-processing techniques were applied in order to boost the quality of images. The data were pre-processed in two stages:

- Image resizing: The images in this dataset have a wide range of dimensions. All of the images that were originally obtained were examined to see if they met the dataset’s minimum height and width requirements. All of the images in the dataset were scaled to this minimum dimension after it was determined. A minimum dimension as obtained in our work is 224 × 224. So, to fit to the input image size in AlexNet and Xception pretrained models, all images in the dataset were resized to the dimension of 224 × 224.
- Image normalization: In this work, contrast enhancement and image normalization methods were utilized to adjust pixel intensity values in order to provide a better-enhanced image in this study. Hidden information that occurs inside the low range is revealed by changing the pixel intensity. As a result, we normalized all image intensity values to a range of [–1, 1]. Given the fact that this method helps remove bias and achieve uniform distribution, it helps in accelerating the convergence of the model.

### 4.3. Data Augmentation

Data augmentation is a common deep learning technique that increases the amount of available training/testing data [15]. The main reason for applying multiple image augmentation techniques is to improve the system’s overall performance through the adding of more diversified data to the already existing limited dataset. Given the small number of positive COVID-19 cases, this is especially critical at this stage. The variation in the original number of the acquired images in every image class can be clearly seen in Table 2. This difference in image count creates a significant class divide. This imbalance in image class might result in various issues that may include overfitting, where the model does not succeed in effectively generalizing the unseen dataset. Data augmentation (rotation and zoom) was used in this article in order to resolve the problem of overfitting that is caused by the limited number of training images. We randomly rotated images by 30 degrees and randomly zoomed them by 20%. Table 2 illustrates the number of images before the data augmentation method (15,153) and after it (26,383). Later, we divided this dataset both data into 80% for training and 20% for validation. Table 3 summarizes the partitions of this data set.

### 4.4. Deep Feature Concatenation (DFC)

**Figure 5:**
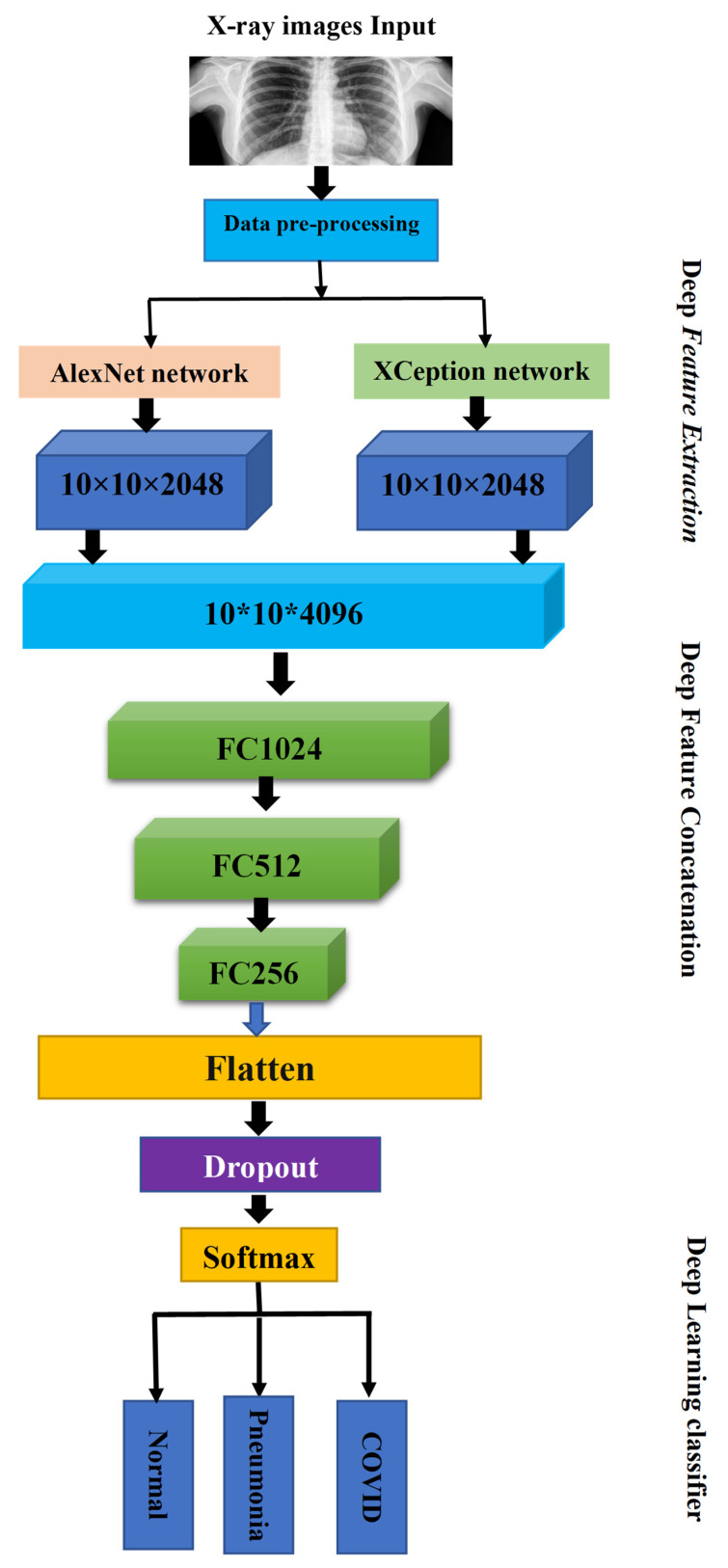
Graphical abstract of proposed method.

To detect COVID-19 from X-rays images, AlexNet and Xception have been used separately by many researchers. In most works, these models give weak results. However, the most challenging difficulty in this study is the relatively limited number of accessible infected images. To overcome this problem in this presented paper, the deep feature concatenation mechanism was used in order to improve the classification process. Firstly, 2 CNNCs have been used to conduct the process of deep feature extraction. We used AlexNet and Xception deep learning models to extract features from X-ray images. The collected features were then combined into a single descriptor for classification using the DFC approach. The principle of DFC is taking a set of different learners and combining them using new learning techniques. Later, three fully connected layers of sizes 1024, 512, and 256 neurons, respectively, were added. Outputs from fully connected layers are unnormalized values. The final action that terminates this model is Softmax activation function layer whose aim is to generate outputs. With Softmax activation function, output values are normalized and turned into probability values. In the case of normal, pneumonia and COVID-19, there are 3 classes in our last classification layer. The architecture of the proposed COVID-AleXception model is shown in Figure 5.

## 5. Implementation and Testbed

In this section, we evaluate the efficiency of the suggested scheme based on the results of the conducted experiments. The Intel Core i7 3.5 GHz processor machine with 16 GB RAM and a 2 GB integrated NVIDIA graphics card was used to train and test all of the methods stated in Section 4. Anaconda3 (Python 3.7) was used to implement image pre-processing algorithms, data augmentation tasks, and deep learning models. The dataset presented in Section 4.1 is divided into two groups: training (80%) and validation (20%). The first is for the training process, while the second is for the final evaluation testing.

The performance of the developed model was analyzed using the following metrics: accuracy, precision, recall, and F1 score. Each measure is delineated as follows:

❖ Accuracy: The accuracy is the percentage of properly predicted images out of the total number of predictions [24]. The accuracy is calculated as:

$$Accuracy=\frac{Number\;of\;correct\;predictions}{Total\;number\;of\;predictions}$$

❖ Precision: The ratio of correctly predicted positive results (TP) to the total number of positive results (TP + FP) forecasted by the model is the accuracy metric. The vast number of FPs results in a lower precision [30]. The precision range is between 0 and 1 and is calculated as follows:

$$Precision=\frac{Number\;of\;True\;Positives}{Number\;of\;True\;Positives+Number\;of\;False\;Positives}$$

❖ Recall: The recall is utilized to measure the right positive forecasts by calculating the ratio between the number of true positive results (TP) to the total number of samples (TP + FN) [30]. The recall is calculated using the following equation:

$$Recall=\frac{Number\;of\;True\;Positives}{Number\;of\;True\;Positives+Number\;of\;False\;Negative}$$

❖ F1 score: The F1 score is one of the metrics used to measure and evaluate the model’s performance. The weighted harmonic between the accuracy and recall is used to compute the F1 score. Its definition is as follows:

$$F1\;score=2\times \frac{Recall\times Precision}{Recall+Precision}$$

where TP—True Positive, FP—False Positive, TN—True Negative, FN—False Negative.

### 5.1. Performance Analysis and Analysis

**Figure 6:**
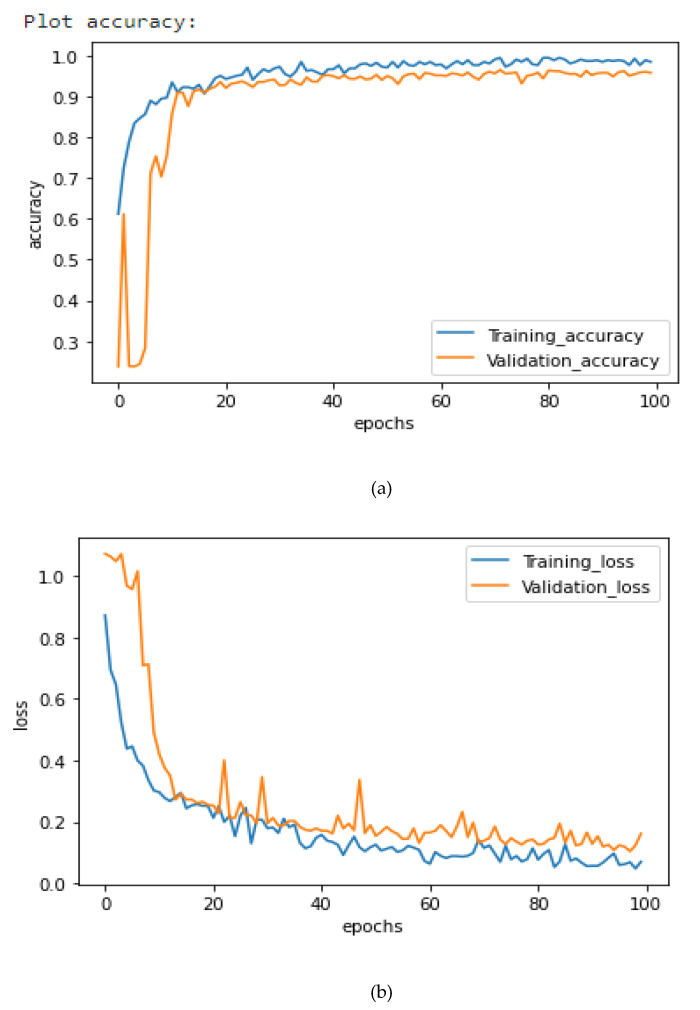
AlexNet Loss and Accuracy curves. (**a**) Training and Validation Accuracy, (**b**) Training and Validation Loss.

**Figure 7:**
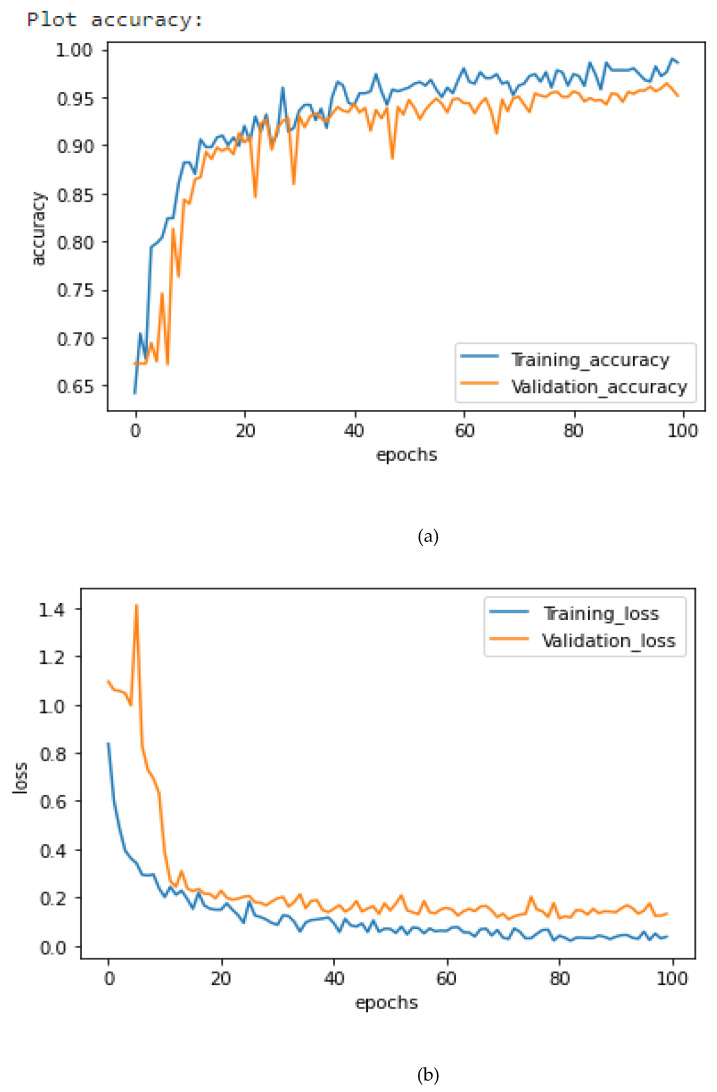
Xception Loss and Accuracy curves. (**a**) Training and Validation Accuracy, (**b**) Training and Validation Loss.

**Figure 8:**
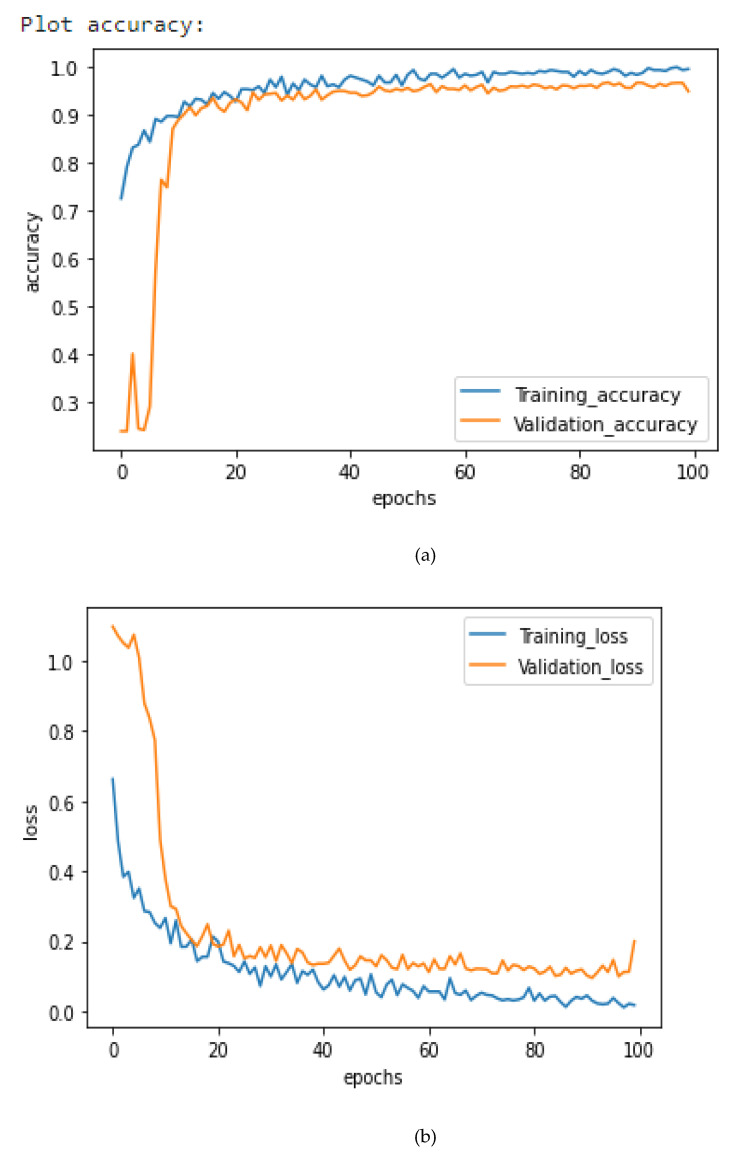
COVID-AleXception Loss and Accuracy curves. (**a**) Training and Validation Accuracy, (**b**) Training and Validation Loss.

Figure 6, Figure 7 and Figure 8 illustrate the suggested models’ model accuracy and loss graphs. As shown in Figure 6, after 100 epochs of validation, the accuracy of the AlexNet model achieves the highest of 94.86%, while the training accuracy reaches 95.25% and the loss is 0.2. As illustrated in Figure 7, after 100 epochs of validation, the accuracy of the Xception model achieves 95.63%, while the training accuracy for this model reaches 96.75% and the loss is 0.16. Concatenating AlexNet and Xception enabled us to get the best result, previous models passing them through the fully connected layer. As it is shown in Figure 8, the use of this hybrid strategy helped us to obtain 98.68% accuracy and only 0.1% loss.

The average results for the classification task in the dataset from the three deep learning models are displayed in Table 4. The training as well as the testing run times are reported second. As it is clearly shown in Table 4, AlexNet and Xception give an average accuracy > 96%. The COVID-AleXception model helped us to achieve the highest average accuracy of 98.68%. The same model gives the highest F1 score equal to 98.46% as well as the finest recall equal to 98.77%. Therefore, the method proposed in this article plays a significant role in evaluating the feasibility and reliability of the detection of COVID-19. However, due to the complicated structures of internal modules, the COVID-AleXception model requires a longer time for training and testing if compared to other models, which is one of the shortcomings of our suggested model. Thus, different computing environments have crucial impacts on the time consumption. Added to that, high-performance hardware is required in order to process these models. 

Table 5, on the other hand, illustrates the classification results for the different classes (normal, pneumonia and COVID-19) which were obtained from the same AlexNet, Xception and COVID-AleXception models for the determination of accuracy, precision, recall and F1 score. As it is presented in the table, AlexNet achieved an accuracy of 96.25%, which is the highest for the normal category. Besides, Xception achieved a recall equal to 97.25%, the highest for the normal category. However, it can be stated that COVID-AleXception attained the precision of 98.55%, the finest F1 score of 98.75% and the recall of 98.72 for COVID-19 class. 

**Figure 9:**
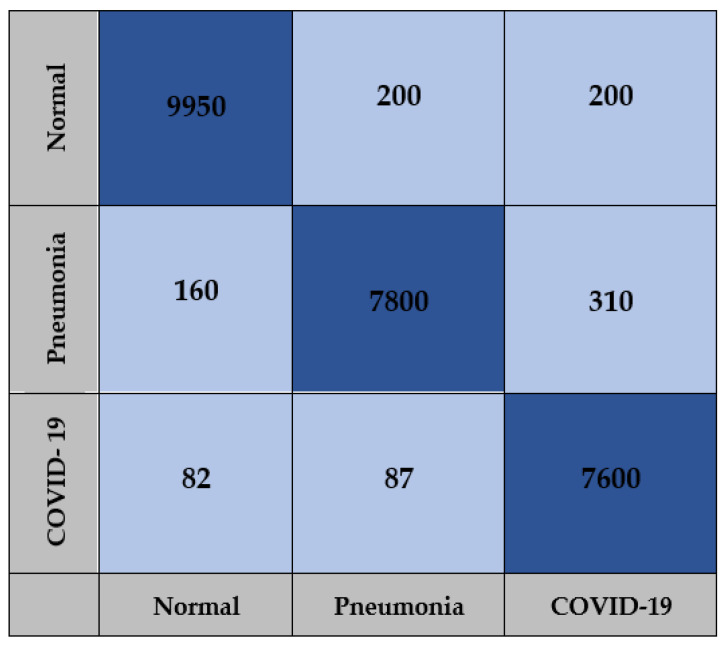
Confusion matrix for AlexNet model.

**Figure 10:**
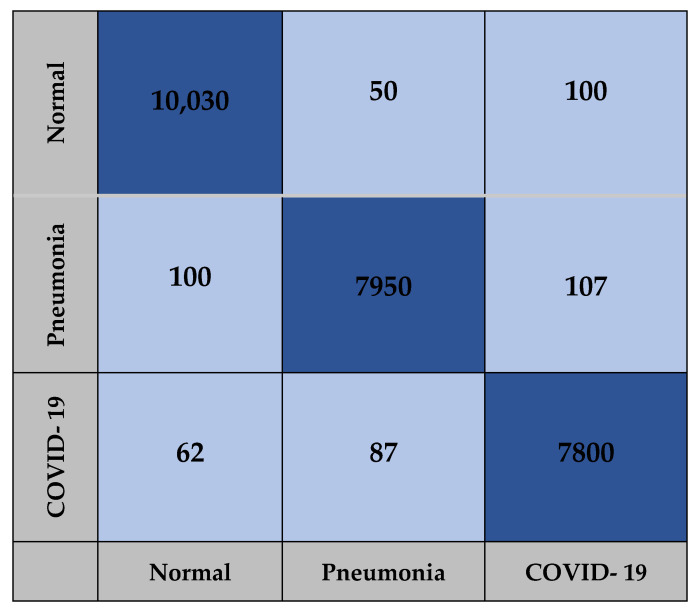
Confusion matrix for Xception model.

**Figure 11:**
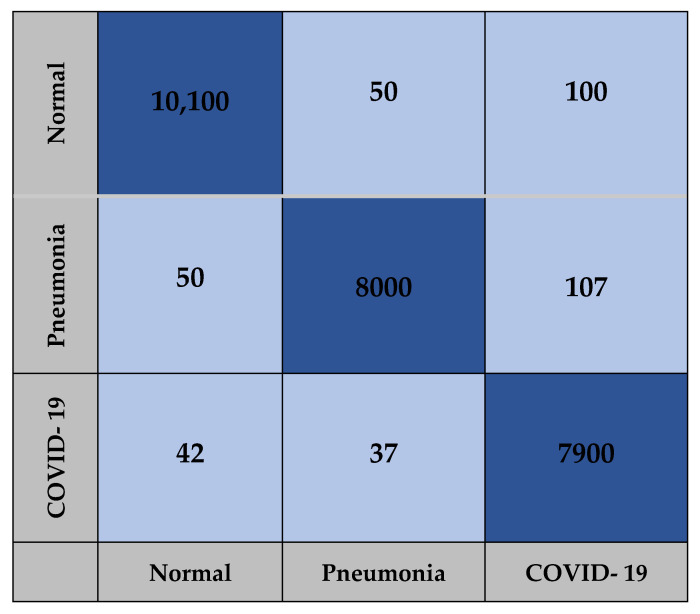
Confusion matrix for COVID-AleXception model.

The confusion matrix for the AlexNet, Xception and COVID-AleXception models used in the study include the findings obtained from training carried out with the test dataset, as presented in Figure 9, Figure 10 and Figure 11. 

### 5.2. Comparative Analysis and Discussion 

The collected results indicated the superiority of our suggested model in terms of accuracy for the three-class classification task when compared to state-of-the-art approaches presented in Table 6. The performance of the COVID-AleXception model is better than the AlexNet and Xception models. The algorithms that were proposed in the state-of-the-art are focused on accuracy, but they don’t take into account the amount of computation time that is required for something such as the training procedure. Although the image classification methods provide essential features for CNN but reduce the complexity of its computation, the amount of time necessary to process would be quite substantial. When training on a huge database, the time needed can indeed be complex, and applications should always wait until training has been completed. COVID-AleXception was developed to ensure that the preparation is completed in the shortest amount of time possible. These results should only be used for reference because the methodologies and metrics used to calculate each system are differ such that an accurate comparison cannot be made.

## 6. Conclusions

Because it spreads through human contact, COVID-19, a severe virus, has had an impact on many nations worldwide. With the ever-increasing demand for PCR tests commonly used to screen for all possible cases of COVID-19, the unavailability of these tests and the emergence of significant false negative rates, developing an alternative diagnostic tool is paramount to minimize risks and spread. In this article, two recent pre-trained CNN models were used to combine features collected from chest X-ray images, utilizing the deep feature concatenation (DFC) technique. For the overall enhancement of the pandemic’s prediction capability, we propose COVID-AleXception: a neural network that concatenates the AlexNet and Xception networks. Several X-ray images were carefully collected from various sources to form a relatively large-scale X-ray image dataset for examining this suggested model. The COVID-AleXception model has a classification accuracy of 98.68%, demonstrating its superiority over AlexNet and Xception, which have classification accuracies of 94.86% and 95.63%, respectively. Since nothing can be perfect, our proposed MobiRes-Net Model has some limitations. One of them is the higher training and testing run times if compared to other models because of the complex structure of the models inside the modules. Another limitation is that high-performance hardware is required in order to process those models. In future, the COVID-AleXception model can be installed in the FPGA (Field-Programmable Gate Array) processor to make a standalone device, which will be easier to integrate with a drone monitoring system.

## Figures and Tables

**Table 2:** Number of images of the training dataset before and after the augmentation technique.

Name of Class	Normal	Pneumonia	COVID-19
No. of images before augmentation	10,192	1345	3616
No. of images after augmentation	10,192	8087	8107

**Table 3:** Details of Train–test class-wise distribution of datasets after the augmentation technique.

Classes	Training (80%)	Validation (20%)	Total
Normal	8154	2038	10,192
Pneumonia	6470	1617	8087
COVID-19	6486	1621	8107

**Table 4:** Average classification results for the classification task of the original dataset.

Model	Accuracy (%)	Precision (%)	Recall (%)	F1-Score (%)	Training Time (s)	Testing Time (s)
AlexNet	94.86	94.75	94.08	94.78	690	3.29
Xception	95.63	94.26	94.12	95.16	740	2.49
COVID-AleXception	98.68	99.11	98.77	98.46	938	4.23

**Table 5:** Model classification results for a 3-category classification task for the original dataset.

Model	Category	Accuracy (%)	Precision (%)	Recall (%)	F1-Score (%)
AlexNet	Normal	96.25	95.25	95.55	95.36
	Pneumonia	95.25	95.45	95.85	95.57
	COVID-19	93.55	93.75	93.35	93.03
Xception	Normal	97.15	97.52	97.25	97.55
	Pneumonia	95.52	95.63	95.25	95.85
	COVID-19	95.01	95.21	95.15	95.05
COVID-AleXception	Normal	99.05	99.35	99.27	99.78
	Pneumonia	98.75	99.85	99.48	99.05
	COVID-19	98.55	98.45	98.72	98.75

**Table 6:** The results obtained compared to state-of-the-art methods.

Ref	CNN Model	Data Sources	Accuracy (%)	Precision (%)	Recall (%)	F1-Score (%)
[15]	nCOVnet	Cohen et al. [25]	97.97	97.61	97.78	97.67
[16]	ResNet50	Radiography Database [26]	99.17	99.45	98.97	99.25
[17]	ResNet18	CT scan images [27]	99.60	99.52	99.71	99.75
	ResNet50		99.20	99.05	99.35	99.25
	ResNet101		99.30	99.35	99.25	99.18
	SqueezeNet		99.50	99.45	99.40	99.55
[18]	VGG19 +CNN	GitHub+ cancer X-ray and CT images [28]	98.05	97.95	97.87	97.90
	ResNet152V2		95.31	95.23	95.17	95.25
	ResNet152V2 + GRU		96.09	95.85	95.95	95.80
	ResNet152V2+ Bi-GRU		93.36	93.15	93.22	93.25
[19]	ResNet50	Cohen [25] Kaggle [29]	93.01	92.90	92.95	92.85
	ResNet101		97.22	97.05	97.15	97.03
[20]	VGG-16	Khan et al. [22] Ozturk [20]	79.58	79.58	85.43	87.49
[21]	FocusCOVID	Kaggle-1 [30] Kaggle-2 [31]	95.20	95.36	95.16	95.26
[22]	CoroNet	Chest X-ray Images [32]	89.6	89.45	89.36	89.50
[23]	CheXImageNet	Cohen [25] Kaggle [29]	100	100	100	100
[24]	ResNet50	Kaggle chest X-ray [33] RSNA pneumonia [34]	91.13	92.96	92.85	92.73
	MobileNet		93.73	93.66	93.55	93.48
	Hybrid model		94.43	94.26	94.35	94.15
COVID-AleXception	Xception	Ref: [25,26,30,31,32]	94.86	94.75	94.08	94.78
	AlexNet		95.63	94.26	94.12	95.16
	COVID-AleXception		98.68	99.11	98.77	98.46

## Data Availability

The datasets used during the current study are available from the corresponding author on reasonable request.
